# Supplementary material for: The HEDGEHOG-GLI1 pathway is important for fibroproliferative properties in keloids and as a candidate therapeutic target
Source: Commun Biol. 2023 Dec 7;6:1235. doi: 10.1038/s42003-023-05561-z (PMC10703807; doi:10.1038/s42003-023-05561-z)
Supplement: Supplementary file 2 — Supplementary Information [file 42003_2023_5561_MOESM2_ESM.pdf]

The HEDGEHOG-GLI1 pathway is important for fibroproliferative properties in keloids and as a candidate therapeutic target

Mamiko Tosa, Yoshinori Abe, Seiko Egawa, Tomoka Hatakeyama, Chihiro Iwaguro, Ryotaro Mitsugi, Ayaka Moriyama, Takumi Sano, Rei Ogawa, Nobuyuki Tanaka

SUPPLEMENTARY INFORMATION

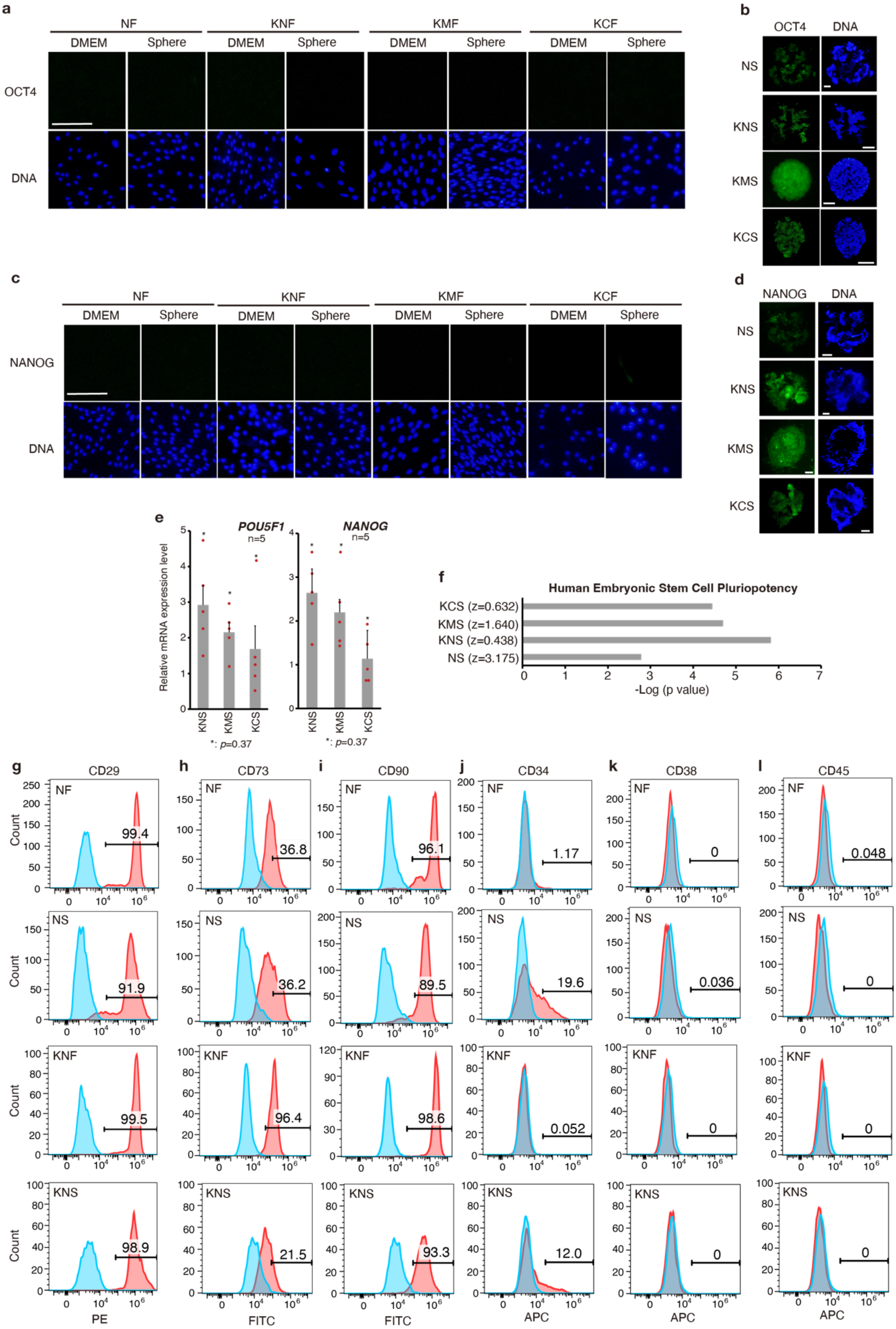
a
NF
KNF
KMF
KCF
DMEM
Sphere
OCT4
DNA
b
OCT4
DNA
NS
KNS
KMS
KCS
c
NANOG
d
e
POU5F1
n=5
NANOG
n=5
Relative mRNA expression level
KNS
KMS
KCS
*: p=0.37
f
Human Embryonic Stem Cell Pluriopotency
KCS (z=0.632)
KMS (z=1.640)
KNS (z=0.438)
NS (z=3.175)
-Log (p value)
g
CD29
h
CD73
i
CD90
j
CD34
k
CD38
l
CD45
Count
99.4
36.8
96.1
1.17
0
0.048
91.9
36.2
89.5
19.6
0.036
0
99.5
96.4
98.6
0.052
0
0
98.9
21.5
93.3
12.0
0
0
PE
FITC
FITC
APC
APC
APC

m

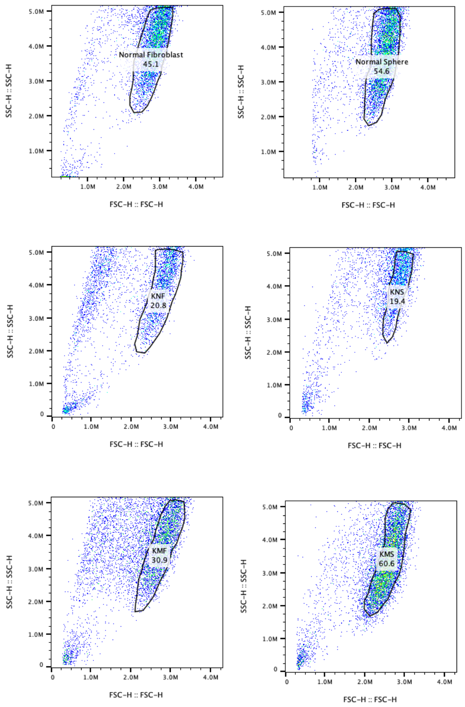

Supplementary Fig. 1. Sphere-forming cells from keloid fibroblasts exhibit stem cell properties.

a, c Representative immunofluorescent image for OCT4 (a) or NANOG (b) expression in keloid fibroblasts (K28) or normal fibroblasts (N3). Fibroblasts were cultured with DMEM hi-glucose supplemented with 10% FBS (DMEM) or sphere culture medium (sphere: DMEM:F-12K [3:1] supplemented with B-27 supplement, 40 ng/ml bFGF, and 20 ng/ml EGF). Fibroblasts were cultured with sphere culture medium for 5 days. NF: transcriptome profiling of normal fibroblasts, KNF: fibroblasts from normal-looking skin adjacent keloids, KMF: keloid marginal area, KCF: keloid central area, NS: normal dermis, KNS: keloid normal dermis, KMS: keloid marginal area, and KCS: keloid central area. **b, d** Representative immunofluorescent images for OCT4 (b) or NANOG (d) expression in keloid fibroblasts or normal fibroblast-derived sphere-forming cells. Green indicates OCT4 or NANOG, and blue indicates DNA (Hoechst33342 staining). Bar, 100 μm. **e** qPCR analysis showing relative expression of stemness genes (*POU5F1* [OCT4], and *NANOG*) in keloid fibroblast–derived stem-like cells (POU5F1, NANOG: K2, K3, K4, K5 and K6) compared with normal dermal fibroblast-derived stem-like cells (n=1). Results are shown as mean ± SD from five patients. **f** Pathway analysis from the gene expression profile of sphere-forming cells predicted upregulation of the signaling pathway for human embryonic stem cell pluripotency. **g–l** Population of cells harboring mesenchymal stem cell properties in keloid fibroblasts (K29), keloid fibroblast-derived spheres, normal

fibroblasts (N6), and normal fibroblast-derived spheres. Blue: isotype control-derived count, red: antibody-derived count. **m** The gating strategy shown in g–l. Patient information is shown in Supplementary Table 1. Source data are provided as Source Data File 2.

a

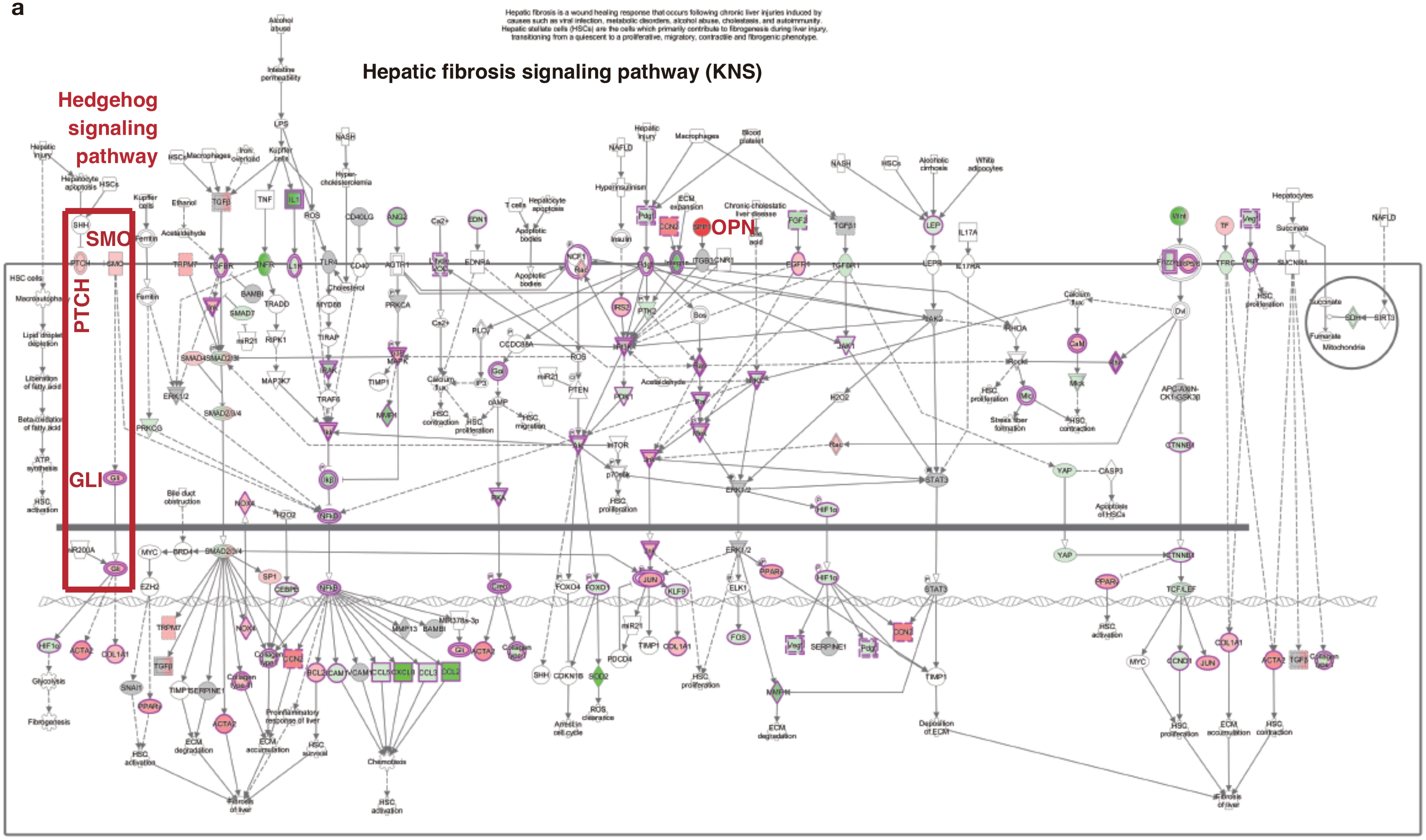

© 2000-2020 QIAGEN. All rights reserved.

b **Osteoathritis pathway (KMS)**

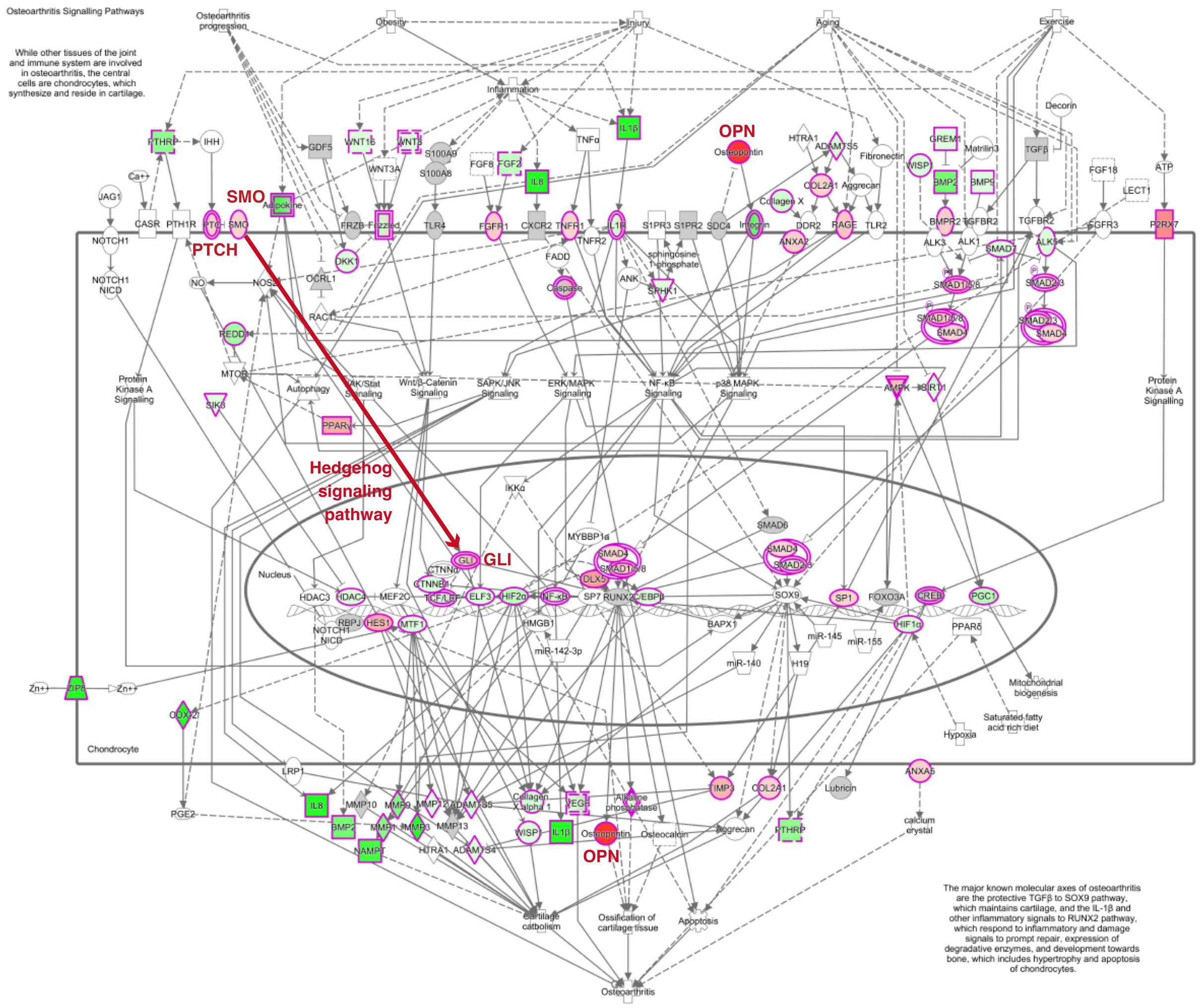

c **Molecular mechanisms of cancer (KNS)**

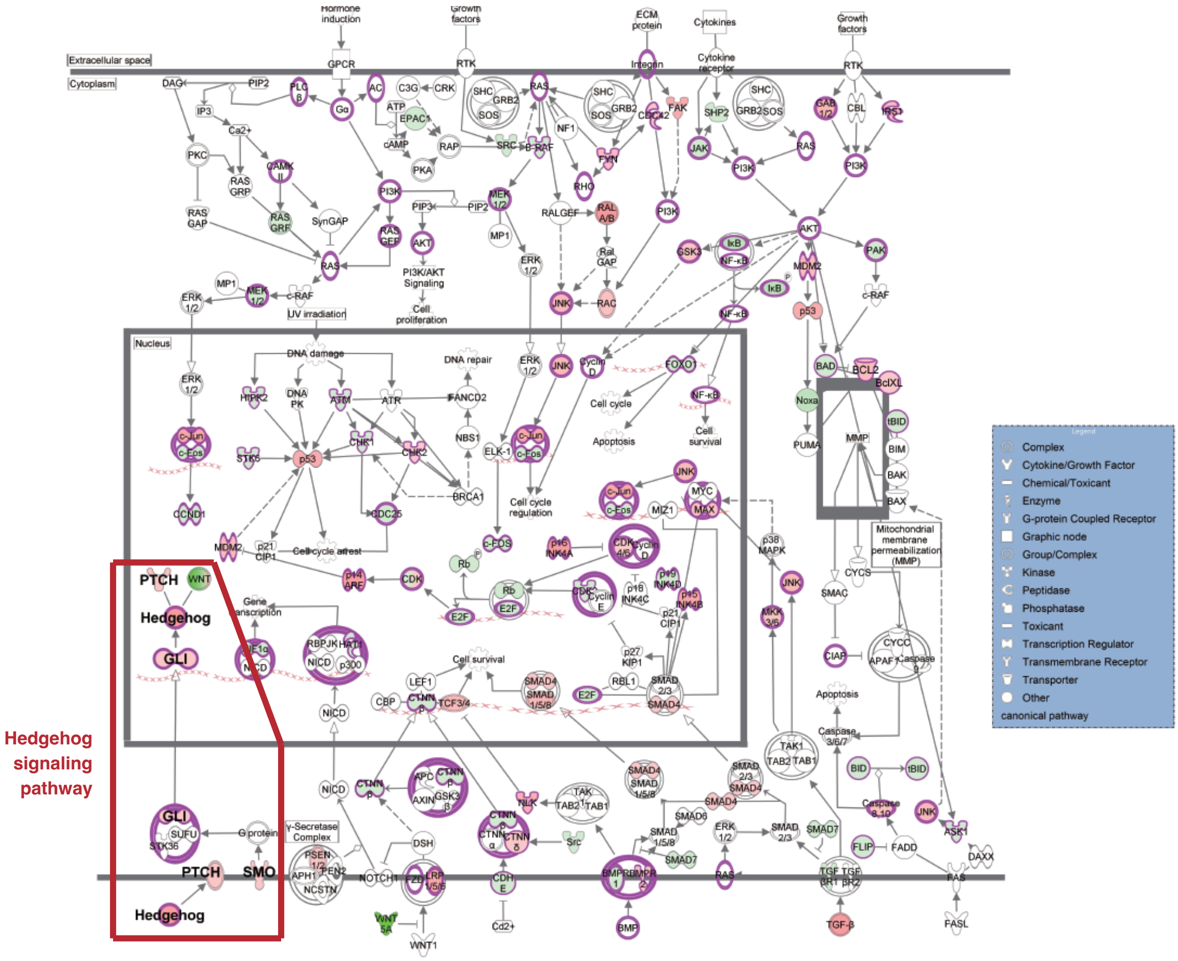

d **Axonal guidance (KNS)**

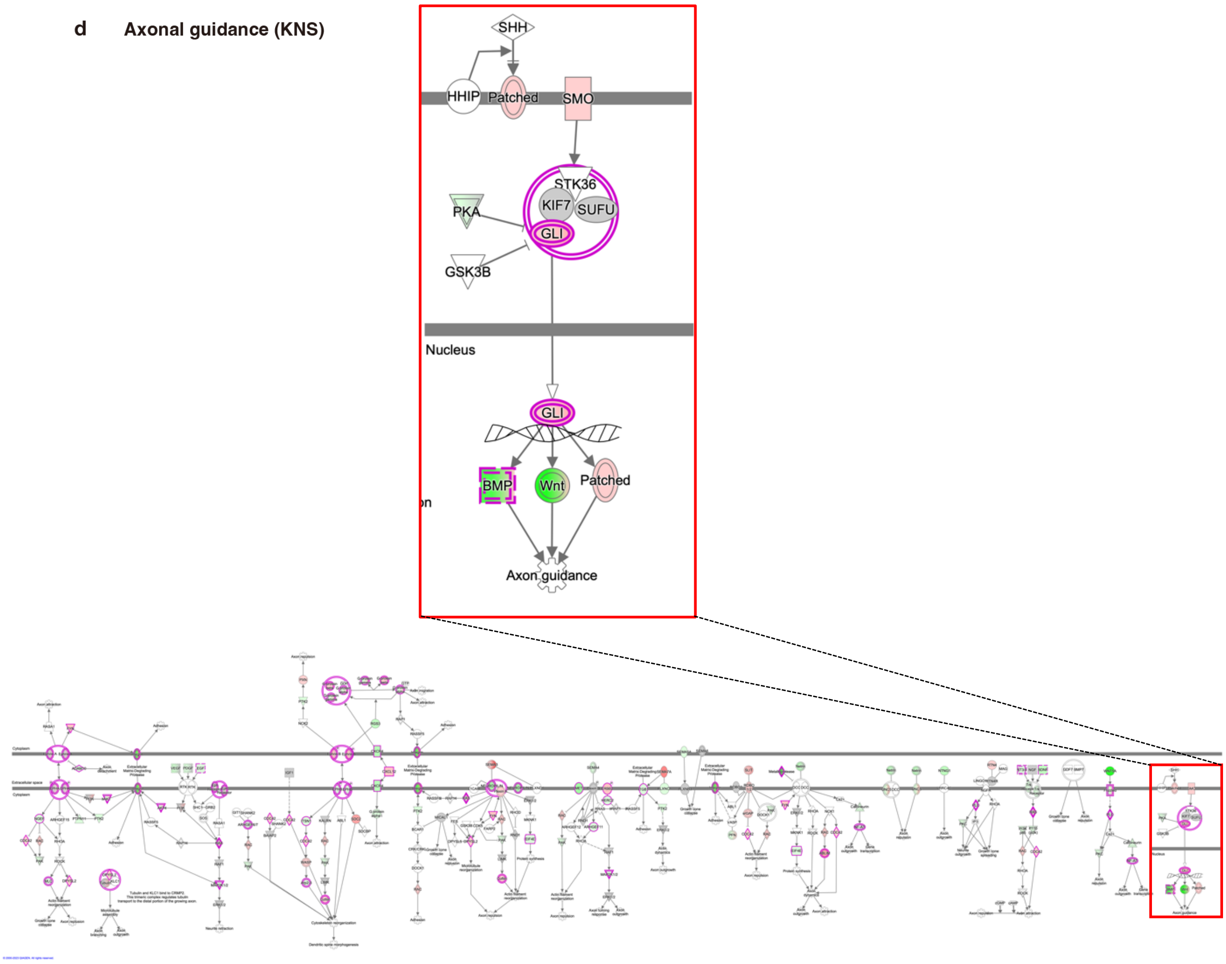

Supplementary Fig. 2. Activated HH signaling pathway–associated canonical pathways from IPA.

a–d IPA revealed that components of the HH signaling pathway are commonly upregulated across canonical pathways associated with hepatic fibrosis (**a**), osteoarthritis (**b**), cancer (**c**), and axonal guidance signaling (**d**). Upregulated genes are shown in red; downregulated genes are shown in green.

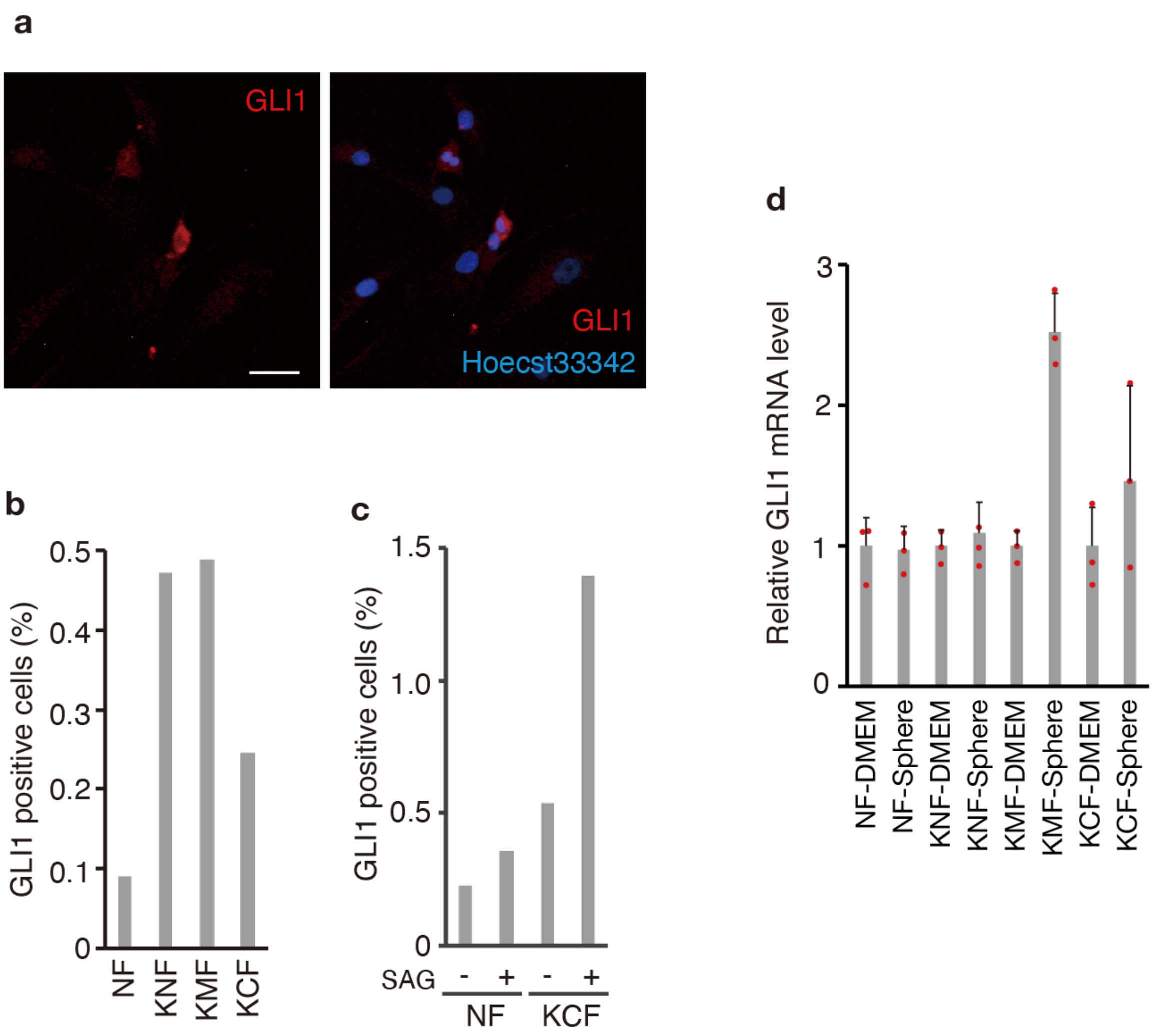

Supplementary Fig. 3. GLI1 expression is abundant in keloid fibroblasts.

a Representative immunofluorescent image of GLI1-expressing fibroblasts (KMF: patient K2). Red indicates GLI1, blue indicates DNA (Hoechst33342 staining). Bar, 200 μm. **b** Quantification of GLI1-positive cells in keloid-derived fibroblast (patient K2). **c** Quantification of GLI1 positive cells in HH signaling pathway–activated cells (patient K2). When cells reached 100% confluency, cells were treated with a SMO agonist, SAG (final 300 nM), for 48 h in DMEM (4.5 mg/ml glucose) supplemented with 0.2% FBS. **d** qPCR analysis for GLI1 mRNA expression level in normal fibroblasts (N3) and keloid fibroblasts (K28) cultured with DMEM hi-glucose supplemented with 10% FBS (DMEM) or sphere culture medium (sphere: DMEM:F-12K [3:1] supplemented with B-27 supplement, 40 ng/ml bFGF, and 20 ng/ml EGF). The results are shown as the mean ± SD from triplicate experiments. Patient information is shown in Supplementary Table 1; the cell counting result and qPCR analysis source data are described in Source Data File 2. NF:

transcriptome profiling of normal fibroblasts, KNF: fibroblasts from normal-looking skin adjacent keloids, KMF: keloid marginal area, and KCF: keloid central area.

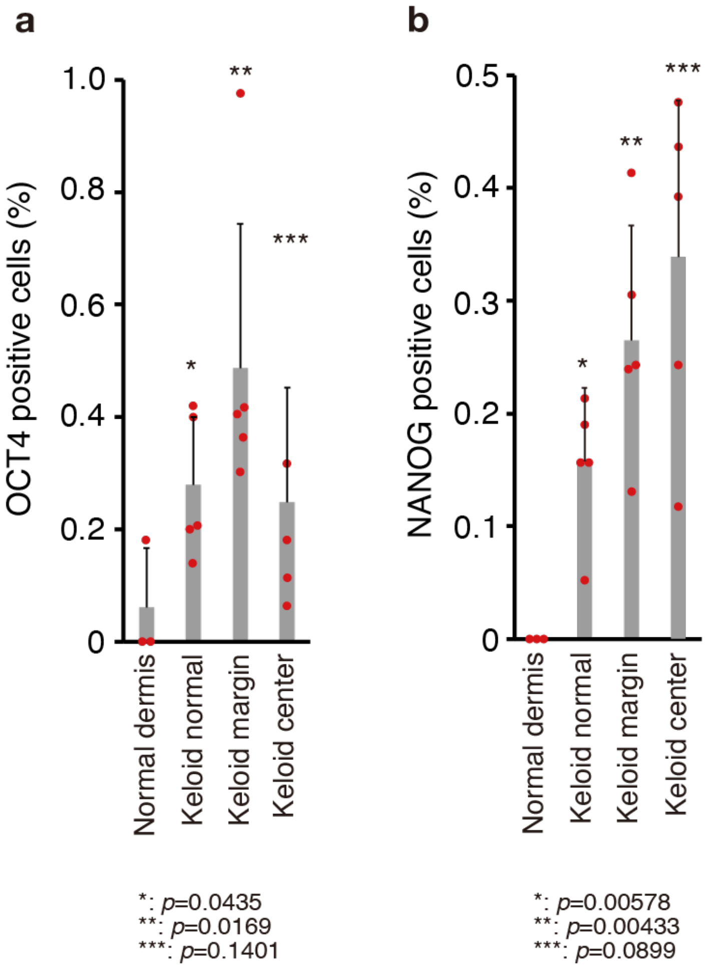

Supplementary Fig. 4. OCT4 and NANOG expression are more abundant in keloid fibroblast–derived stem cells than in normal fibroblast–derived stem-like cells.

a, b Quantification of OCT4 (**b**) and NANOG (**c**) -positive cells from Fig. 2c–f. Results are shown as the mean ± SD from five patient–derived keloid fibroblasts (K2, K3, K4, K5, K6) and one donor-derived normal fibroblast (N5). Patient information is shown in Supplementary Table 1; source data are provided as Source Data File 2.

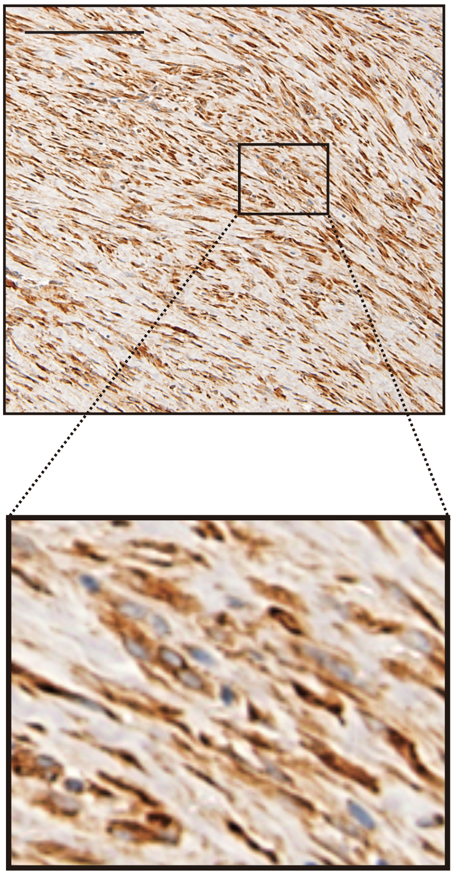

Supplementary Fig. 5. SHH expression in keloid tissue, related to Figure 3.
Magnified image of SHH-positive area in keloid tissue (K9). Bar, 200 μm.

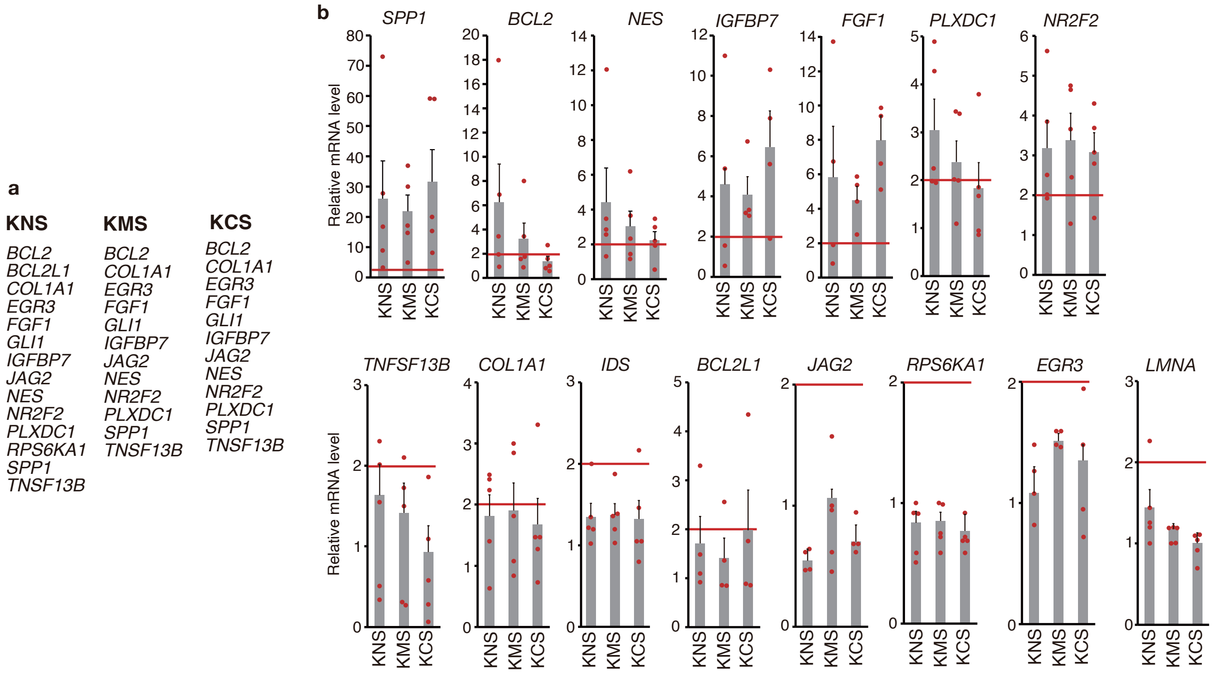

Supplementary Fig. 6. Candidate GLI1-mediated upregulated gene expression in keloid fibroblast–derived stem-like cells.

a Putative GLI1-mediated upregulated genes in keloid fibroblast–derived stem cells from IPA. **b** qPCR analysis revealed that 7 of 15 genes shown in **a** were upregulated in keloid fibroblast–derived stem cells (K1, K3, K7, K10 [n=4], fold change versus stem cells from normal dermis-derived fibroblast [N1]. Fold expression change >2.0 compared with NS is GLI1-mediated upregulated genes in keloid fibroblast–derived stem cells. KNS, KMS, and KCS were described in Fig. 1. Results are shown as means ± SD. Patient information is shown in Supplementary Table 1; source data are provided as Source Data File 2. KNS: keloid normal dermis, KMS: keloid marginal area, and KCS: keloid central area.

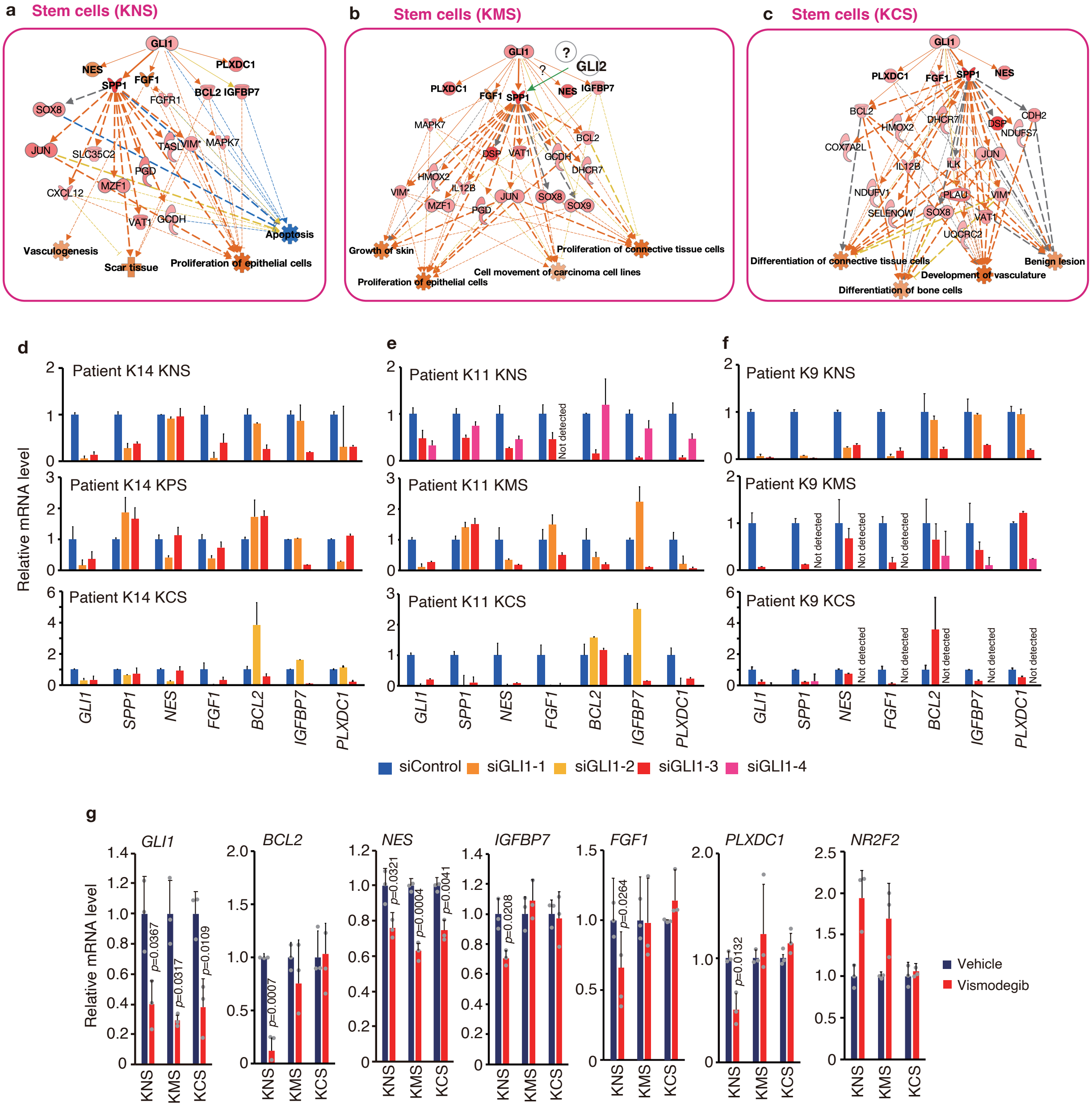

Supplementary Fig. 7. Roles of GLI-mediated upregulated genes in keloid fibroblast–derived stem cells from three areas.

a–c IPA revealed that GLI1 and its target genes provide site-specific function in keloid fibroblast–derived stem cells. GLI1 target genes are also shown in Supplementary Fig. 5a, b. **d–f** qPCR analysis showing validation of enhanced expression of putative GLI1 target genes in keloid fibroblast-derived stem cells. siRNAs for GLI1 (siGLI1-1, siGLI1-2, siGLI1-3, siGLI1-4) were stably expressed by recombinant lentivirus. GLI1-knocked-down cells were obtained by puromycin selection. Cells (300,000, passage 2) were seeded in a 6 cm ultra-low attachment dish and incubated for 10 days. Data are shown

as means ± SD from three patient–derived keloid stem cells (K14, K11, K9). **g** qPCR analysis reveals HH signaling pathway–mediated GLI1 target genes in keloid stem cells. Cells (10,000, patient K14, passage 2) were seeded in 6-well ultra-low attachment plate and incubated for four days. Next, 10 μM vismodegib was treated for four days. Results are shown as means ± SD from triplicated experiments. Patient information is shown in Supplementary Table 1; source data are provided as Source Data File 2. KNS (Keloid normal), KMS (keloid marginal area), and KCS (keloid central area) are described in Fig. 1.

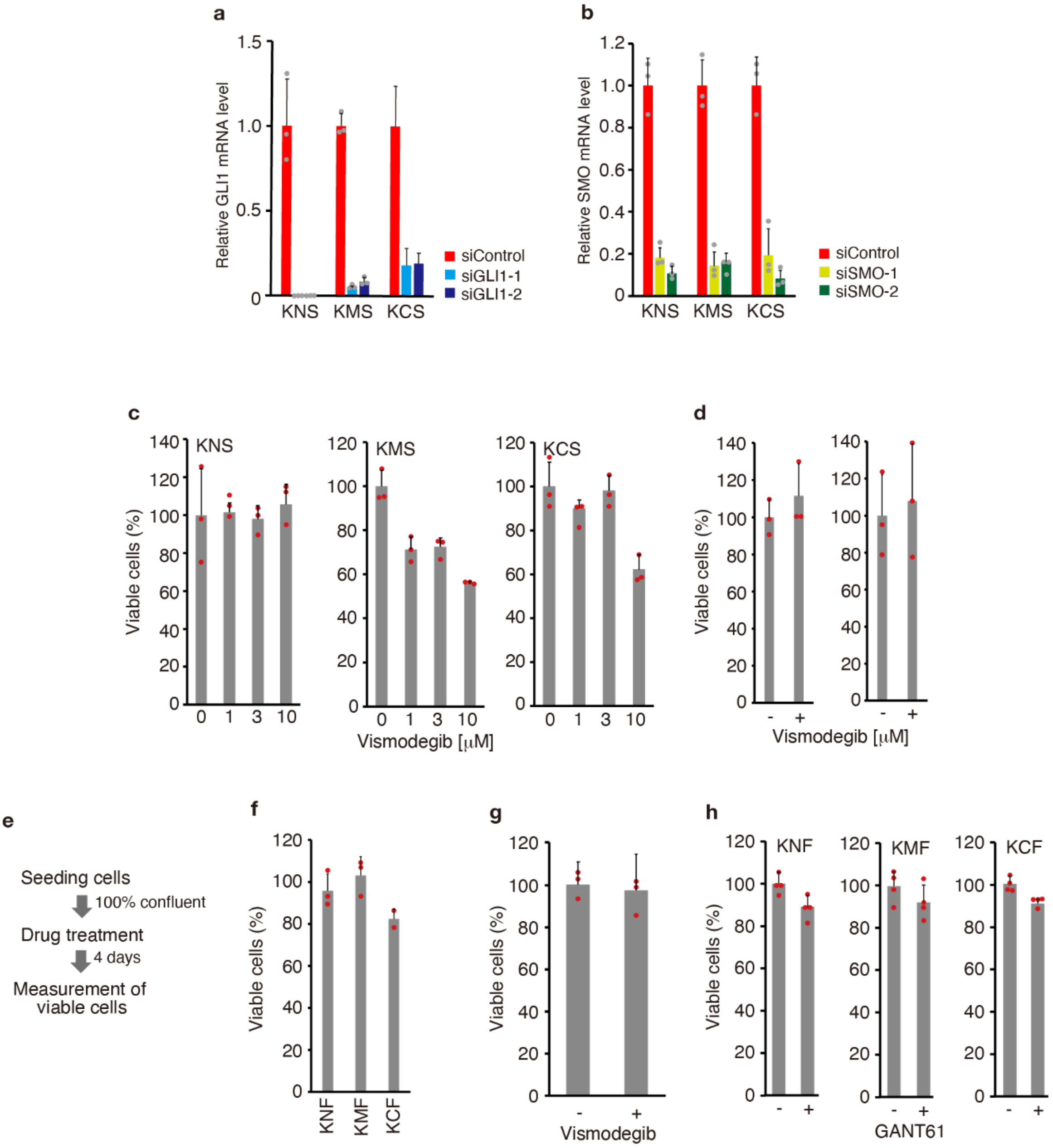

Supplementary Fig. 8. Effect of vismodegib for keloid fibroblast–derived stem cells, keloid fibroblasts, and normal fibroblasts.

a, b qPCR analysis showing *GLI1* (**a**) or *SMO* (**b**) expression level in RNAi-mediated GLI1 (**a**) or SMO (**b**) knocked down keloid fibroblast–derived stem cells (patient K14). siRNAs for GLI1 (siGLI1-1, siGLI1-2) or siSMO (siSMO-1, siSMO-2) were stably expressed by recombinant lentivirus. GLI1-knocked down stem cells were also used for the experiment shown in Fig. 4. Results are shown as means ± SD. **c** Sensitivity to SMO inhibitor among keloid fibroblast–derived stem cells from three keloid areas. The experimental design for drug treatment is shown in Fig. 5a. Viable cells were quantified using the Cell Counting Kit-8. **d** SMO inhibitor treatment does not affect stem cells from

normal dermis-derived fibroblasts (N1, N5). **e** Schematic illustration of the experimental design in **f–h. f, g** SMO inhibitor treatment (10 μM) does not affect fibroblasts from keloid tissue (**f**: n=3 [patients K16, K17, K18]) as well as the normal dermis (**g**: N1). **h** GLI inhibitor treatment (10 μM) does not affect fibroblasts from keloid tissue (K15). Viable cells were quantified as described in **c**. Patient information is shown in Supplementary Table 1; source data are provided as Source Data File 2. KNS: keloid normal dermis, KMS: keloid marginal area, and KCS: keloid central area.

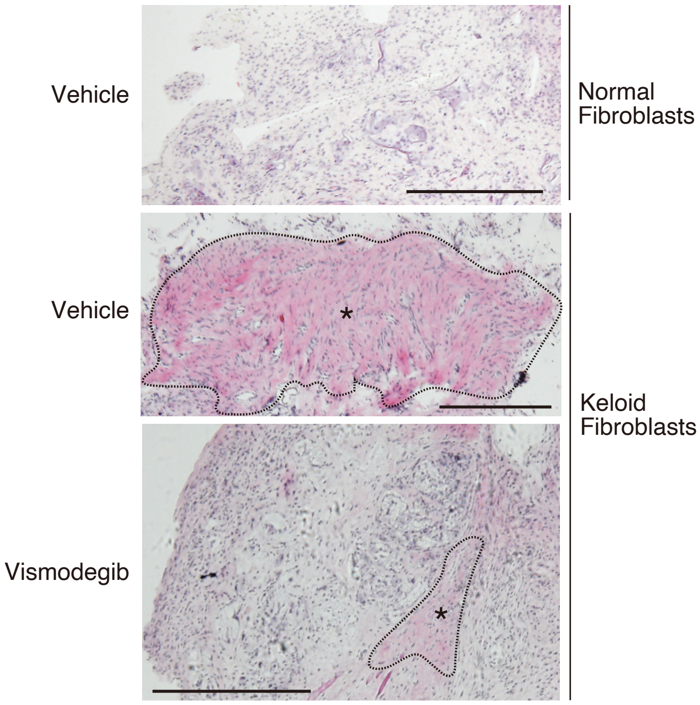

Supplementary Figure 9. Representative H&E staining image of keloid fibroblast transplant with or without vismodegib treatment.

The asterisk shows the keloid bundle area: bar, 500 μm.

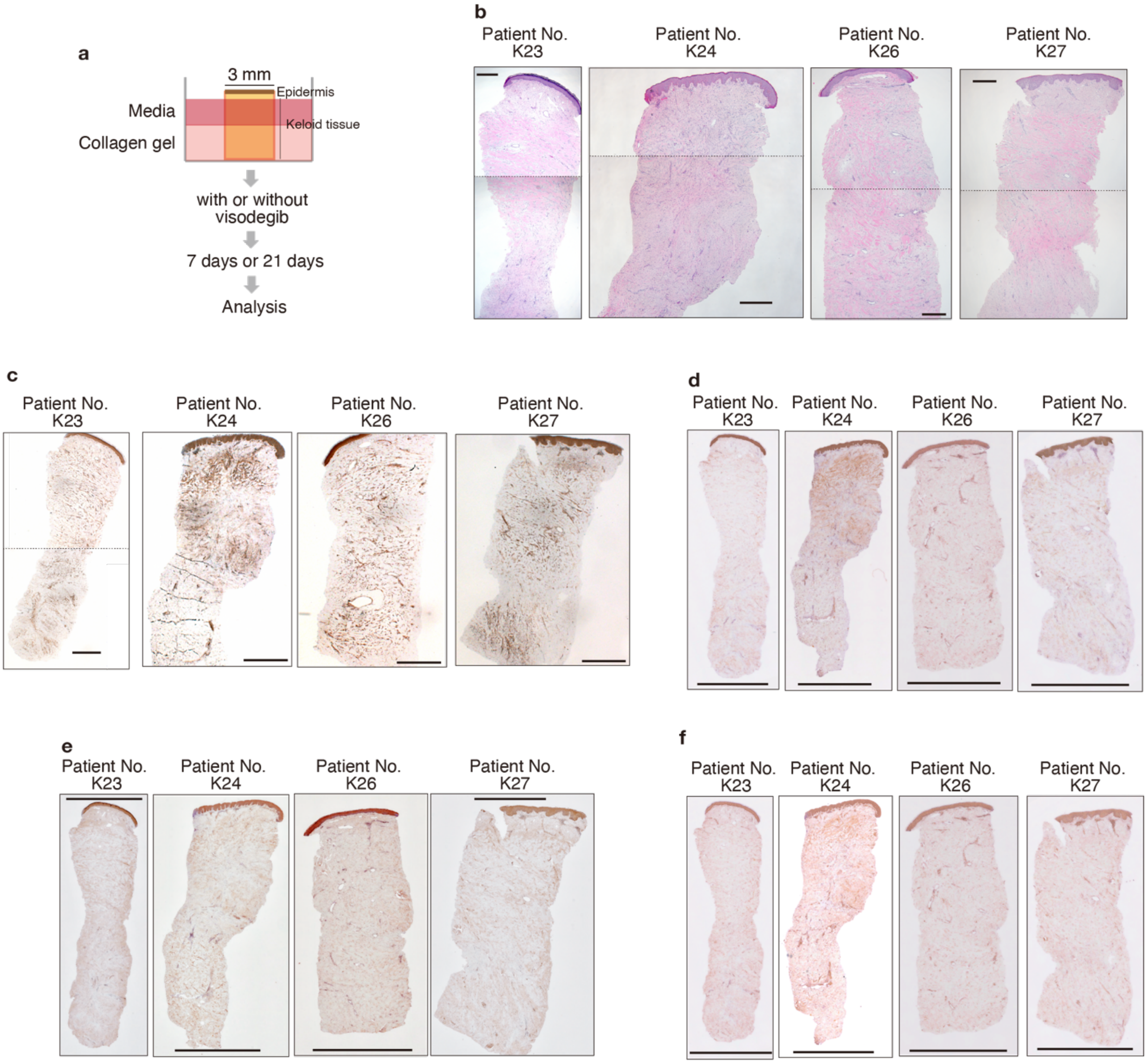

Supplementary Figure 10. Expression of keloid-associated molecules in keloid organ culture.

a Schematic illustration of the experimental design for keloid ex vivo culture and drug treatment. **b** H&E staining to visualize collagen bundles in keloid organ culture. **c–e** Expression of keloid-associated molecules (c: IL-6, d: β-catenin, e: TGF-β. f: SMAD3). Bar, 500 (b, c) or 2500 μm. Patient information is shown in Supplementary Table 1.

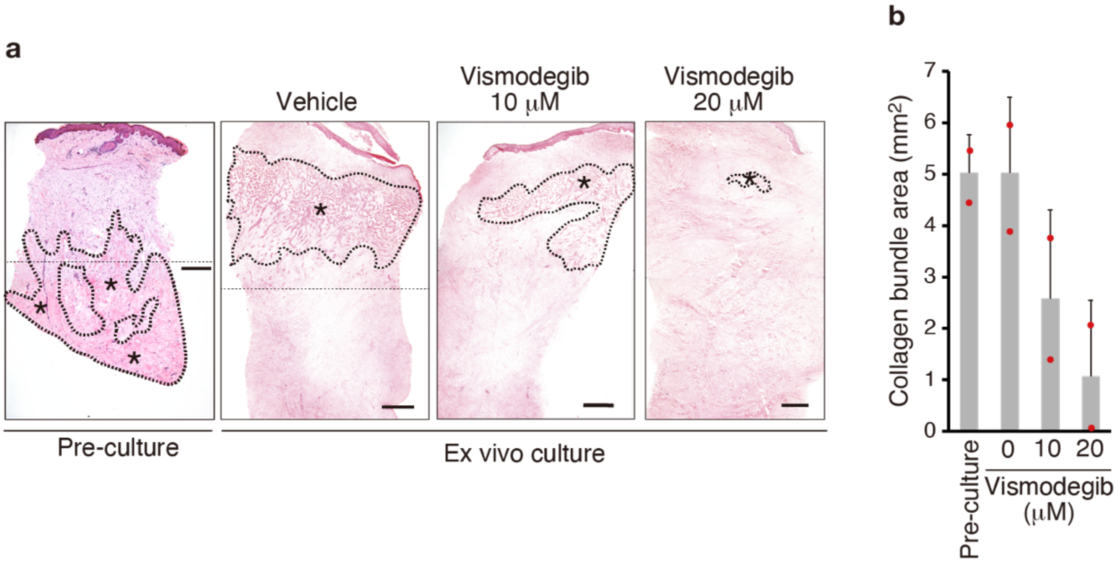

Supplementary Figure 11. Long-term vismodegib treatment in keloid organ culture.

a Representative H&E staining of keloid organ culture with or without vismodegib treatment. Bar, 500 μm. The asterisk shows the keloid bundle area. Vismodegib was treated for 21 days at the indicated dose. **b** Quantification of keloid bundle area with or without vismodegib treatment in keloid organ culture. Results are shown as the mean ± SEM from two patients (patients K22, K23). The dot represents each collagen bundle area from each patient-derived keloid organ culture. Patient information is shown in Supplementary Table 1; source data are provided as a Source Data File 2. Bar, 1000 μm.

a

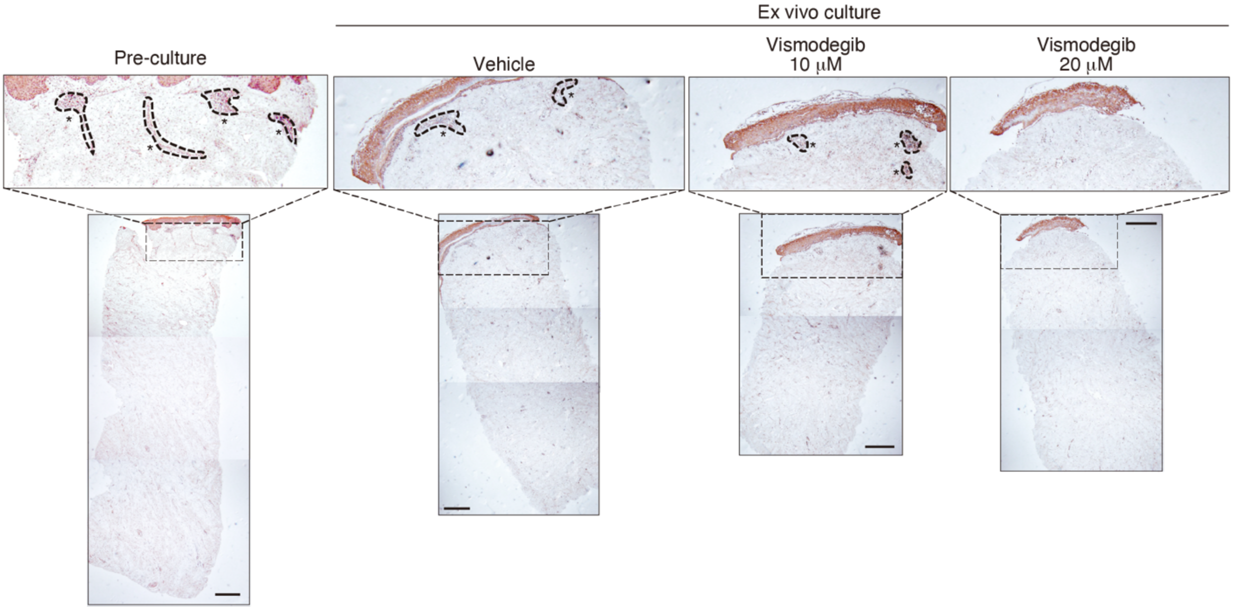
Ex vivo culture
Pre-culture
Vehicle
Vismodegib
10 μM
Vismodegib
20 μM

b

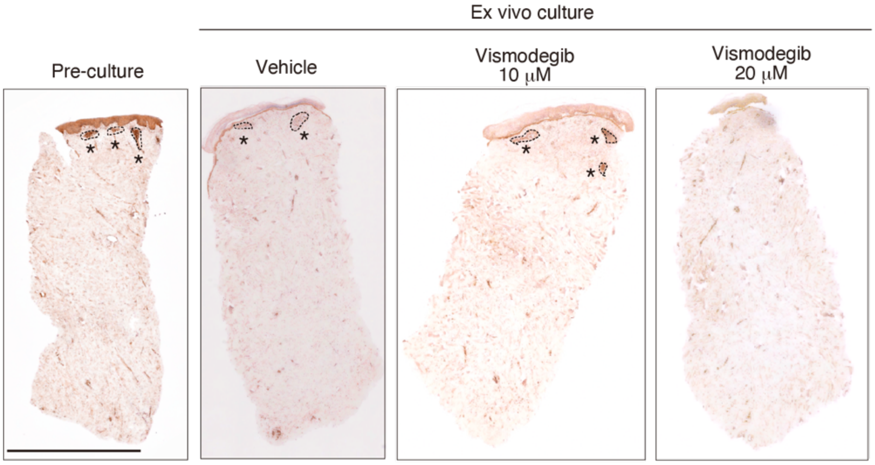
Ex vivo culture
Pre-culture
Vehicle
Vismodegib
10 μM
Vismodegib
20 μM

c

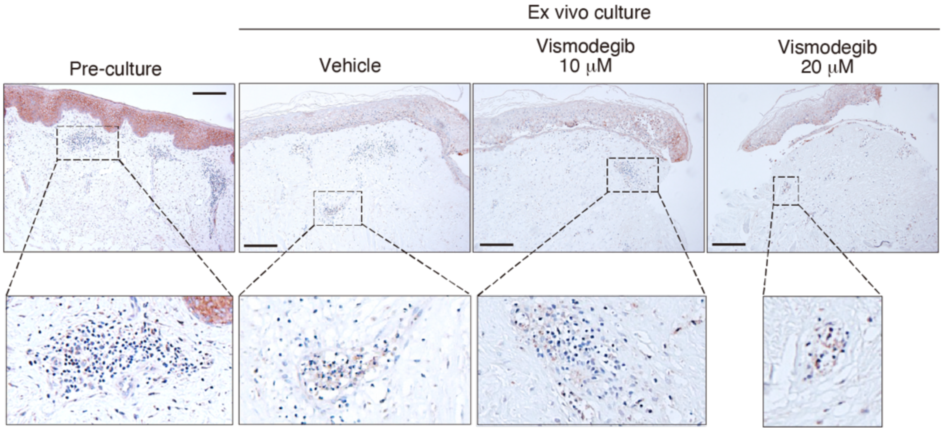
Ex vivo culture
Pre-culture
Vehicle
Vismodegib
10 μM
Vismodegib
20 μM

Supplementary Figure 12. TGF-β expression and β-CATENIN localization after vismodegib treatment in keloid organ culture.

a, **b** TGF-β (a) or β-CATENIN (b) staining of keloid organ culture with or without vismodegib treatment (Patient K27). Bar, 500 (a) or 1000 μm (b). In a or b, the asterisk shows the TGF-β-expressing area (a) or β-CATENIN-expressing area. Vismodegib treatment was applied for 7 days at the indicated dose. Patient information is shown in Supplementary Table 2.

Supplementary Table 1: Patient information

Sample ID		Age	Sex	Site	Analysis
Patient	K1	36	Male	Chest	qPCR analysis for GLI1 expression (Fig2 a–c)<br>IHC analysis for SHH expression (Fig3 d)<br>IHC analysis for OPN expression (Fig4 f, i)<br>qPCR analysis for GLI1-mediated upregulated genes expression (FigS6 b)
	K2	31	Female	Shoulder	qPCR analysis for GLI1 expression (Fig2 c)<br>Quantification of GLI1 and OCT4 positive cells (Fig2 e, g)<br>IHC analysis for SHH expression (Fig3 d)<br>IHC analysis for OPN expression (Fig4 f, i)<br>qPCR analysis for POU5F1 and NANOG expression (Fig. S1e)<br>Quantification of OCT4 and NANOG positive cells (Fig.S4 a,b)<br>IHC analysis of GLI1-expressing keloid fibroblasts (FigS3 a-c)
	K3	57	Male	Chest	Quantification of GLI1 and OCT4 positive cells (Fig2 g)<br>Quantification of OCT4 and NANOG positive cells (Fig.S4 a,b)<br>qPCR analysis for PTCH1 expression (Fig3 f)<br>IHC analysis for SHH expression (Fig3 d)<br>IHC analysis for OPN expression (Fig4 f, i)<br>qPCR analysis for POU5F1 and NANOG expression (Fig. S1e)<br>qPCR analysis for GLI1-mediated upregulated genes expression (FigS6 b)
	K4	30	Female	Lower abdomen	qPCR analysis for GLI1 expression (Fig2 c)<br>Quantification of GLI1 and OCT4 positive cells (Fig2 g)<br>Quantification of OCT4 and NANOG positive cells (Fig2 b, c)<br>IHC analysis for SHH expression (Fig3 d)<br>IHC analysis for OPN expression (Fig4 f, i)<br>qPCR analysis for POU5F1 and NANOG expression (Fig. S1e)<br>Quantification of OCT4 and NANOG positive cells (Fig.S4 a,b)
	K5	55	Female	Chest	qPCR analysis for GLI1 expression (Fig2 c)<br>Quantification of GLI1 and OCT4 positive cells (Fig2 g)<br>qPCR analysis for PTCH1 expression (Fig3 f)<br>qPCR analysis for POU5F1 and NANOG expression (Fig. S1e)<br>Quantification of OCT4 and NANOG positive cells (Fig.S4 a,b)
	K6	63	Female	Chest	qPCR analysis for GLI1 expression (Fig2 c)<br>Quantification of GLI1 and OCT4 positive cells (Fig2 g)<br>qPCR analysis for POU5F1 and NANOG expression (Fig. S1e)<br>Quantification of OCT4 and NANOG positive cells (Fig4S a,b)<br>IHC analysis for SHH expression (Fig3 d)
	K7	30	Male	Chest	qPCR analysis for PTCH1 and SMO expression (Fig3 e, h, i)<br>qPCR analysis for GLI1-mediated upregulated genes expression (Fig.S6 b)
	K8	47	Male	Chest	qPCR analysis for GLI1 expression (Fig2 c)<br>IF analysis for co-expression of GLI1 and OCT4 (Fig2 c)<br>qPCR analysis for SPP1 expression (Fig4 e)<br>Quantification of viable stem cells (Fig5 f)
	K9	28	Male	Chest	IF analysis for co-expression of GLI1 and OCT4 (Fig2 c)<br>IHC analysis for SHH expression (Fig3 a d)<br>IHC analysis for OPN expression (Fig4 f, i)<br>qPCR analysis for average suppression level of GLI1 target gene expression (Fig4 d)<br>Quantification of viable stem cells (Fig5 f)<br>IHC analysis for SHH expression (Fig.S5)<br>qPCR analysis of GLI1-mediated upregurated gene (Fig.S 7 f)
	K10	26	Male	Chest	IF analysis for co-expression of GLI1 and OCT4 (Fig2 c)<br>Quantification of viable stem cells (Fig5 f)<br>qPCR analysis for GLI1-mediated upregulated genes expression (Fig.S6 b)
	K11	51	Male	Chest	IF analysis for GLI1, OCT4 and Nanog expression (Fig2 d, f)<br>qPCR analysis for average suppression level of GLI1 target gene expression (Fig4 d)<br>Quantification of viable stem cells (Fig5 f)<br>qPCR analysis of GLI1-mediated upregurated gene (Fig.S 7 e)
	K12	32	Female	Chest	IF analysis for co-expression of GLI1 and OCT4 (Fig2 c)<br>IF analysis for GLI1, OCT4 and Nanog expression (Fig2 d, f)<br>qPCR analysis for PTCH1 expression (Fig3 f)<br>GSEA with SMO inhibitor treatment (Fig6)<br>Quantification of viable stem cells with SMO inhibitor treatment(Fig.S 8 c)

	K13	34	Male	Chest	Quantification of viable stem cells (Fig5 f)<br>Quantification of transplant volume (Fig6 b)<br>GSEA with or without SMO inhibitor treatment (Fig6 d)
	K14	30	Male	Chest	qPCR analysis for suppression level of GLI1 target gene expression (Fig4 d)<br>Quantification of viable stem cells (Fig5 a-f)<br>qPCR analysis for GLI1-mediated upregurated genes (Fig.S 7 d,g)<br>qPCR analysis for GLI1 or SMO (Fig.S8 a,b)
	K15	26	Male	Chest	Quantification of viable stem cells (Fig5 f)<br>Quantification of viable keloid fibroblasts with GLI1 inhibitor treatment (Fig.S8 h)
	K16	34	Female	Lower abdomen	Quantification of viable keloid fibroblasts with SMO inhibitor treatment (Fig.S8 f)
	K17	31	Female	Chest	Quantification of viable stem cells (Fig5 f)<br>Quantification of viable keloid fibroblasts with SMO inhibitor treatment (Fig.S8 f)
	K18	31	Male	Lower abdomen	Quantification of viable stem cells (Fig5 f)<br>Quantification of xenografted transplant volume (Fig6 b)<br>GSEA with SMO inhibitor treatment (Fig6 d)<br>Quantification of viable keloid fibroblasts with SMO inhibitor treatment (Fig.S8 f)
	K19	32	Female	Shoulder	Quantification of viable stem cells (Fig5 f, g)
	K20	38	Male	Chest	Quantification of viable stem cells (Fig5 f)
	K21	33	Male	Chest	qPCR analysis for IL6, CTGF and COL1A2 expression (Fig6 c)
	K22	28	Male	Ear	HE staining to visualize collagen bundle in keloid organ culture (Fig7 a. b)<br>HE staining to visualize collagen bundle in keloid organ culture with long-term vismodegib treatment (Fig.S11 a,b)
	K23	22	Male	Ear	HE staining to visualize collagen bundle in keloid organ culture (Fig7 a. b)<br>IHC analysis for IL6 expression (Fig7 c, d)<br>IHC analysis for CTGF expression (Fig7 e, f)<br>IHC analysis for OPN expression (Fig7 g, h)<br>HE staining to visualize collagen bundle in keloid organ culture with vismodegib treatment (Fig.S9)<br>Quantification of collagen bandle area with long-term vismodegib treatment (Fig.S 11 b)<br>HE and IHC analysis for keloid-associated molecules expression (Fig.S10 b-f)
	K24	26	Male	Ear	HE staining to visualize collagen bundle in keloid organ culture (Fig7 a. b)<br>HE and IHC analysis for keloid-associated molecules expression (Fig.S10 b-f)
	K25	26	Female	Shoulder	HE staining to visualize collagen bundle in keloid organ culture (Fig7 a. b)
	K26	22	Male	Ear	IHC analysis for IL6 expression (Fig7 c, d)<br>IHC analysis for CTGF expression (Fig7 e, f)<br>IHC analysis for OPN expression (Fig7 g, h)<br>HE and IHC analysis for keloid-associated molecules expression (Fig.S10 b-f)
	K27	24	Male	Ear	IHC analysis for IL6 expression (Fig7 c, d)<br>IHC analysis for CTGF expression (Fig7 e, f)<br>IHC analysis for OPN expression (Fig7 g, h)<br>HE and IHC analysis for keloid-associated molecules expression (Fig.S10 b-f)<br>IHC analysis for TGF-β and β-catenin expression (Fig.S12 a, b)
	K28	32	Male	Shoulder	IF analysis for OCT4 and NANOG expression (Fig.S1 a-d)<br>qPCR analysis for GLI1 expression (Fig.S3 d)
	K29	29	Female	Chest	FCM analysis for mesenchymal stem cells property in keloid fibroblasts (Fig.S1 g-l)
Normal skin	N1	31	Male	Chest	qPCR analysis for GLI1-mediated upregulated genes expression (Fig.S6 b)<br>Quantification of viable keloid fibroblasts with GLI1 inhibitor treatment (Fig.S8 d, g)
	N2	28	Male	Abdomen	Quantification of GLI1 and OCT4 positive cells (Fig2 g)<br>Quantification of xenografted transplant volume (Fig6 b)<br>Quantification of OCT4 and NANOG positive cells (Fig2 b, c)
	N3	36	Female	Chest	Quantification of GLI1 and OCT4 positive cells (Fig2 g)<br>Quantification of OCT4 and NANOG positive cells (Fig2 b, c)<br>IF analysis for OCT4 and NANOG expression (Fig.S1 a-d)<br>qPCR analysis for GLI1 expression (Fig.S3 d)

	N4	24	Female	Upper arm	IHC analysis of GLI1-expressing fibroblasts (Fig.S3 a-c)<br>HE staining to visualize collagen bundle in xenografted transplant with vismodegib treatment (Fig.S9)
	N5	42	Male	Chest	Quantification of viable keloid fibroblasts with GLI1 inhibitor treatment (Fig.S8 d)
	N6	26	Male	Chest	FCM analysis for mesenchymal stem cells property in keloid fibroblasts (Fig.S1 g-l)
Mature Scar	MS1	22	Female	Shoulder	IHC analysis for SHH expression (Fig3 c, d)<br>IHC analysis for OPN expression (Fig4 f)

Supplementary Table 2: shRNA sequences for GLI1 and SMO

siRNA	Sequence
siGLI1-1	Sense–CCGGGCCTTCTGCCACCAAGCTAACTCGAGTTAGCTTGGTGGCAGAAGGTTTTTG<br>Anti-sense–AATTCAAAAACCTTCTGCCACCAAGCTAACTCGAGTTAGCTTGGTGGCAGAAGGC
siGLI1-2	Sense–CCGGGCAGTAAAGCCTTCAGCAACTCGAGTTGCTGAAGGCTTTACTGCTTTTTG<br>Anti-sense–AATTCAAAAAGCAGTAAAGCCTTCAGCAACTCGAGTTGCTGAAGGCTTTACTGC
siGLI1-3	Sense–CCGGGCCAACTTGCCCAATCACAACTCGAGTTGTGATTGGGCAAGTTGGTTTTTG<br>Anti-sense–AATTCAAAAACCAACTTGCCCAATCACAACTCGAGTTGTGATTGGGCAAGTTGGC
siGLI1-4	Sense–CCGGGCAAATAGGGCTTCACATACTCGAGTATGTGAAGCCCTATTTGCTTTTTG<br>Anti-sense–AATTCAAAAAGCAAATAGGGCTTCACATACTCGAGTATGTGAAGCCCTATTTGC
siSMO-1	Sense–CCGGGTCATGACTCTGTTCTCCATCTCGAGATGGAGAACAGAGTCATGA TTTTTG<br>Anti-sense–AATTCAAAAATCATGACTCTGTTCTCCATCTCGAGATGGAGAACAGAGTCATGAC
siSMO-2	Sense–CCGGGCTGCCACTTCTACGACTTCTCGAGAAGTCGTAGAAGTGGCAGC TTTTTG<br>Anti-sense–AATTCAAAAAGCTGCCACTTCTACGACTTCTCGAGAAGTCGTAGAAGTGGCAGC

Supplementary Table 3: qPCR probe list

Gene symbol	Assay ID
BCL2	Hs0498639_m1
BCL2L1	Hs00236329_m1
COL1A1	Hs00164004_m1
COL1A2	Hs01028956_m1
CTGF	Hs00170014_m1
EGR3	Hs04935588_m1
FGF1	Hs01092738_m1
GLI1	Hs00171790_m1
GLI2	Hs01119974_m1
GLI3	Hs00609233_m1
IDS	Hs01064295_m1
IGFBP7	Hs00266026_m1
IL6	Hs00985639_m1
JAG2	Hs00171432_m1
LMNA	Hs00153462_m1
NES	Hs04187831_m1

NR2F2	Hs00819630_m1
PLXDC1	Hs00964712_m1
RPS6KA1	Hs01546654_m1
SMO	Hs01090242_m1
SPP1	Hs00959010_m1
TNSF13B	Hs00198106_m1
β-actin	Hs01060665_g1

Supplementary Table 4: Primary antibody list

Antibody	Manufacturer	Catalogue No.	Usage	Dilution
IL6	abcam	ab9324	IHC	1:50
CTGF	abcam	ab8448	IHC	1:50
OPN	abcam	ab53281	IHC	1:2000
SHH	abcam	ab6992	IHC	1:1000
GLI1	SANTA CRUZ BIOTECHNOLOGY	sc-515751	IF	1:1000
NANOG	abcam	ab181557	IF	1:500
OCT4	abcam	ab21624	IF	1:300
TGF-β	SANTA CRUZ BIOTECHNOLOGY	Sc-146	IHC	1:100
β-CATENIN	abcam	ab16051	IHC	1:1400
SMAD3	abcam	Ab40854	IHC	1:1000
PE-conjugated IgG1 kappa isotype control	BIOLEGEND	400114	FCM	According to manufucturer's instruction
PE-conjugated CD29 (Clone: TS2/16)	BIOLEGEND	303003	FCM	According to manufucturer's instruction
FITC-conjugated IgG1 kappa isotype control	BIOLEGEND	400110	FCM	According to manufucturer's instruction
FITC-conjugated	BIOLEGEND	344015	FCM	According to manufucturer's

CD73 (Clone: AD2)				instruction
FITC-conjugated CD90 (Clone: 5E10)	BIOLEGEND	328107	FCM	According to manufucturer's instruction
APC-conjugated IgG1 kappa isotype control	BIOLEGEND	400122	FCM	According to manufucturer's instruction
APC-conjugated CD34 (Clone: 581)	BIOLEGEND	343509	FCM	According to manufucturer's instruction
APC-conjugated CD38 (Clone: HB-7)	BIOLEGEND	356605	FCM	According to manufucturer's instruction
APC-conjugated CD45 (Clone: HI30)	BIOLEGEND	304011	FCM	According to manufucturer's instruction

Supplementary Table 5: Secondary antibody list

Antibody	Manufacturer	Catalogue No.	Usage	Dilution
Histofine® SimpleStain Rat MAX-PO (MULTI)	Nichirei Biosciences	414191	IHC	One drop
Alexa488	Thermo Fisher	A11001	IF	1:1000
Alexa546	Thermo Fisher	A11035	IF	1:1000
