# Supplementary material for: The HEDGEHOG-GLI1 pathway is important for fibroproliferative properties in keloids and as a candidate therapeutic target
Source: Commun Biol. 2023 Dec 7;6:1235. doi: 10.1038/s42003-023-05561-z (PMC10703807; doi:10.1038/s42003-023-05561-z)
Supplement: Supplementary file 3 — Description of additional supplementary Files [file 42003_2023_5561_MOESM3_ESM.docx]

**Description of additional supplementary Files**

**File name:** Source Data File 1

**Description:** The source data of the graph in Figure 1–7.

**File name:** Source Data File 2
**Description:** The source data of the graph in Supplementary Figure 1–12.
