# Supplementary material for: The HEDGEHOG-GLI1 pathway is important for fibroproliferative properties in keloids and as a candidate therapeutic target
Source: Commun Biol. 2023 Dec 7;6:1235. doi: 10.1038/s42003-023-05561-z (PMC10703807; doi:10.1038/s42003-023-05561-z)
Supplement: Supplementary file 4 — Reporting Summary [file 42003_2023_5561_MOESM4_ESM.pdf]

nature portfolio

Corresponding author(s): Nobuyuki Tanaka

Last updated by author(s): 2023-11-6

Reporting Summary

Nature Portfolio wishes to improve the reproducibility of the work that we publish. This form provides structure for consistency and transparency in reporting. For further information on Nature Portfolio policies, see our Editorial Policies and the Editorial Policy Checklist.

Statistics

For all statistical analyses, confirm that the following items are present in the figure legend, table legend, main text, or Methods section.

n/a	Confirmed	
☐	☒	The exact sample size (*n*) for each experimental group/condition, given as a discrete number and unit of measurement
☐	☒	A statement on whether measurements were taken from distinct samples or whether the same sample was measured repeatedly
☐	☒	The statistical test(s) used AND whether they are one- or two-sided<br>*Only common tests should be described solely by name; describe more complex techniques in the Methods section.*
☒	☐	A description of all covariates tested
☐	☒	A description of any assumptions or corrections, such as tests of normality and adjustment for multiple comparisons
☐	☒	A full description of the statistical parameters including central tendency (e.g. means) or other basic estimates (e.g. regression coefficient) AND variation (e.g. standard deviation) or associated estimates of uncertainty (e.g. confidence intervals)
☐	☒	For null hypothesis testing, the test statistic (e.g. *F*, *t*, *r*) with confidence intervals, effect sizes, degrees of freedom and *P* value noted<br>*Give P values as exact values whenever suitable.*
☒	☐	For Bayesian analysis, information on the choice of priors and Markov chain Monte Carlo settings
☒	☐	For hierarchical and complex designs, identification of the appropriate level for tests and full reporting of outcomes
☒	☐	Estimates of effect sizes (e.g. Cohen's *d*, Pearson's *r*), indicating how they were calculated

Our web collection on statistics for biologists contains articles on many of the points above.

Software and code

Policy information about availability of computer code

Data collection	Microarray data was collected by TAKARABIO (Shiga, Japan) using Gene Chip Scanner 3700 7G.<br>qPCR data was collected by StepOne software v2.2.3.
Data analysis	Microarray data was analyzed with Affymetrix Expression Console Software 1.4.1 offered SST-RMA and SST-Alt Splice Analysis for gene and exon level analysis using Affymetrix default analysis settings. Clariom Array data analysis was performed using Transcriptome Analysis Console (TAC) software 8. Gene pathway analysis were performed using Ingenuity Pathwawy Analysis softwawre (QIAGEN).<br>Gene set enrichment analysis (GSEA) was performed with GSEA software V3.0 (BROAD Institute, MIT).<br>Pathologic images were analysed using ??

For manuscripts utilizing custom algorithms or software that are central to the research but not yet described in published literature, software must be made available to editors and reviewers. We strongly encourage code deposition in a community repository (e.g. GitHub). See the Nature Portfolio guidelines for submitting code & software for further information.

Data

Policy information about availability of data

All manuscripts must include a data availability statement. This statement should provide the following information, where applicable:

- Accession codes, unique identifiers, or web links for publicly available datasets
- A description of any restrictions on data availability
- For clinical datasets or third party data, please ensure that the statement adheres to our policy

Microarray data have been deposited in the Gene Expression Omnibus(GEO) database under accession codes GSE218894 and GSE218922, respectively.
All other relevant data supporting the key findings of this study are available within the article and its Supplementary Information files or from the corresponding author upon reasonable request. Source data are provided with this paper.

Human research participants

Policy information about studies involving human research participants and Sex and Gender in Research.

Reporting on sex and gender	N/A
Population characteristics	All the patients in this research were Japanese. Keloid tissues were harvested during plastic surgery from 27 patients confirmed to have clinical and pathological evidence of keloid (Supplementary Table 1). No patient received chemotherapy, radiotherapy or intralesional steroids treatment prior to sugery. Normal scar tissues were obtained from two patients who underwent elective scar resection surgery (Supplementary Table 1). Normal skins were obtained from five patients who underwent elective mature scar resection surgery (Supplementary Table 1). Keloids and normal scars were diagnosed on the basis of their clinical appearance and pathology.
Recruitment	Participants were recruited in Nippon Medical School Hospital. We considered the characteristics of participants from experimental and control groups and no self-selection bias was present.
Ethics oversight	This study was approved by the Medical and Ethics Committees of Nippon Medical School Hospital and each patient signed an informed consent before enrolling in this study.

Note that full information on the approval of the study protocol must also be provided in the manuscript.

Field-specific reporting

Please select the one below that is the best fit for your research. If you are not sure, read the appropriate sections before making your selection.

☒ Life sciences ☐ Behavioural & social sciences ☐ Ecological, evolutionary & environmental sciences

For a reference copy of the document with all sections, see nature.com/documents/nr-reporting-summary-flat.pdf

Life sciences study design

All studies must disclose on these points even when the disclosure is negative.

Sample size	Sample sizes were not predetermined based on statistical methods, but were chosen according to the standards of the field (at least three independent biological replicates for each condition). Required experimental sample sizes were estimated based on previous established protocols in the field. The sample sizes were adequate as the differences between experimental groups were reproducible. All n values are clearly indicated within the figure legends.
Data exclusions	N/A
Replication	Key experiments were conducted using multiple patients-derived cells or tissues. Detailed information is described in Method section.
Randomization	Randomization was not necessary for the other experiments because randomization was not required for these experiments based on previous experience.
Blinding	Blinding was not required in this study and investigators were not blinded to group allocation as all data in this study were analyzed equivalently within defined experimental groups

Reporting for specific materials, systems and methods

We require information from authors about some types of materials, experimental systems and methods used in many studies. Here, indicate whether each material, system or method listed is relevant to your study. If you are not sure if a list item applies to your research, read the appropriate section before selecting a response.

Materials & experimental systems

n/a	Involved in the study
☐	☒ Antibodies
☒	☐ Eukaryotic cell lines
☒	☐ Palaeontology and archaeology
☐	☒ Animals and other organisms
☒	☐ Clinical data
☒	☐ Dual use research of concern

Methods

n/a	Involved in the study
☒	☐ ChIP-seq
☐	☒ Flow cytometry
☒	☐ MRI-based neuroimaging

Antibodies

Antibodies used

We describe all antibodies used in this study in the MATERIALS AND METHODS part of the manuscript.
1.mouse anti-IL-6, abcam, Cat# ab9324.
2.rabbit anti-CTGF, abcam, Cat# ab6992.
3.rabbit anti-OPN, Abcam, Cat# ab8448.
4.rabbit anti-SHH, Abcam, Cat# ab53281.
5.Rabbit anti-Nanog, Abcam, Cat# ab21624.
6.rabbit anti-OCT4, Abcam, Cat# ab181557.
7.mouse anti-GLI1 Santa Cruz Cat# sc515751.
8.Goat anti-Mouse IgG (H+L) Highly Cross-Adsorbed Secondary Antibody, Alexa Fluor 488, Thermo Fisher, Cat# A-11001.
9.Goat anti-Rabbit IgG (H+L) Highly Cross-Adsorbed Secondary Antibody, Alexa Fluor Plus 546, Thermo Fisher, Cat# A-11035.
10. Rabbit anti-beta Catenin, Abcam, Cat#ab16051
11. Rabbit anti-Smad3, Abcam, Cat#ab40854
12. Rabbit anti TGFβ1, Santa cruz, Cat#sc-146

Validation

1. https://www.abcam.com/il-6-antibody-12-2b11-2g10-ab9324.html
2. http://www.abcam.com/CTGF-antibody-ab6992.html
3. https://www.abcam.com/Osteopontin-antibody-ab8448.html
4. https://www.abcam.com/Sonic-Hedgehog-antibody-EP1190Y-ab53281.html
5. https://www.abcam.com/nanog-antibody-ab21624.html
6. https://www.abcam.com/oct4-antibody-epr17929-chip-grade-ab181557.html
7. https://datasheets.scbt.com/sc-515751.pdf
8. https://www.thermofisher.com/antibody/product/Goat-anti-Mouse-IgG-H-L-Cross-Adsorbed-Secondary-Antibody-Polyclonal/A-11001
9. https://www.thermofisher.com/antibody/product/Goat-anti-Rabbit-IgG-H-L-Highly-Cross-Adsorbed-Secondary-Antibody-Polyclonal/A-11035
10. https://www.abcam.co.jp/products/primary-antibodies/beta-catenin-antibody-ab16051.html
11. https://www.abcam.co.jp/products/primary-antibodies/Smad3-antibody-ab16051.html
12. https://www.scbt.com/tgfbeta1-antibody-sc-146

Animals and other research organisms

Policy information about studies involving animals; ARRIVE guidelines recommended for reporting animal research, and Sex and Gender in Research

Laboratory animals

5 week old male athymic BALB/c-AJcl nu/nu mice obtained from JAPAN CLEA were used in this project. Mice were housed at 18-24°C with 40-70% humidity.

Wild animals

The study did not involve wild animals.

Reporting on sex

N/A

Field-collected samples

N/A

Ethics oversight

All procedures involving mice were conducted in accordance with National Health and Medical Research Council (NHMRC) regulations on the use and care of experimental animals and the study protocol approved by the animal experiment committee at the Nippon Medical School (approval number 29-045).

Note that full information on the approval of the study protocol must also be provided in the manuscript.

Flow Cytometry

Plots

Confirm that:

☒ The axis labels state the marker and fluorochrome used (e.g. CD4-FITC).

☒ The axis scales are clearly visible. Include numbers along axes only for bottom left plot of group (a 'group' is an analysis of identical markers).

☒ All plots are contour plots with outliers or pseudocolor plots.

☒ A numerical value for number of cells or percentage (with statistics) is provided.

Methodology

Sample preparation	The method for flow cytometry analysis was described in the METHODS part. Fibroblasts were rinsed with ice-cold PBS and dissociated from dishes with 2.5% Trypsin solution (Nacalai Tesque). For dissociation of sphere-forming cells, Accumax solution (Nacalai Tesque) was used. After dissociation of cells, cells were washed with ice-cold FCM buffer (2% FBS in PBS) twice. Then cells were stained with isotype control and the cell surface marker.
Instrument	BECKMAN COULTER CytoFlex
Software	Data collection: CytoExpert2.4<br>Data analysis: FlowJo 10
Cell population abundance	N/A
Gating strategy	Preliminary gating was performed with FSC-SSC plot. Then positive/negative cell population were determined by isotype control and replicate separately stained with the cell surface marker.

☐ Tick this box to confirm that a figure exemplifying the gating strategy is provided in the Supplementary Information.
